# Treatment of Noncirrhotic Portal Hypertension Secondary to Portal Vein Thrombosis With Endovascular Mesocaval Shunt Creation

**DOI:** 10.7759/cureus.8086

**Published:** 2020-05-13

**Authors:** Parkpoom Phatharacharukul, Maximilian Pyko, Nabil Fayad

**Affiliations:** 1 Gastroenterology and Hepatology, Indiana University, Indianapolis, USA; 2 Interventional Radiology, Indiana University, Indianapolis, USA

**Keywords:** hemobilia, portal hypertension, portal vein thrombosis, mesocaval shunt, obscure gastrointestinal bleeding

## Abstract

Transjugular intrahepatic portosystemic shunt (TIPS) creation can be very beneficial to decrease high portal pressure and its consequent dreadful complications, such as variceal hemorrhage. However, some anatomical limitations such as portal vein thrombosis can make TIPS technically impossible to perform. Here, we describe a case of a 72-year-old female patient who previously had a Roux-En-Y choledochojejunostomy, which was complicated by portal vein thrombosis. The patient subsequently developed portal hypertension, and this was successfully treated with endovascular mesocaval shunt creation, given that TIPS was not a viable option.

## Introduction

Patients with portal hypertension, both cirrhotic and non-cirrhotic, can develop some serious complications, including variceal hemorrhage and ascites. Treatment modalities include reversing the etiologies of portal hypertension (such as hepatitis C virus [HCV] eradication in patients with HCV cirrhosis, or anticoagulation in patients with portal vein thrombosis), directly decreasing the portal vein pressure, and treating the secondary affects (such as diuretics for ascites, or endoscopic banding of esophageal varices).

Creation of a transjugular intrahepatic portosystemic shunt (TIPS), which is an artificial channel connecting the portal and hepatic venous systems, is an effective treatment for portal hypertension [1]. However, TIPS is not always technically feasible due to anatomical inaccessibility, such as in patients with complete portal vein thrombosis.

In these cases, percutaneous mesocaval shunt creation, which connects the mesenteric vessels directly to the systemic circulation through the inferior vena cava, is an alternative treatment technique that can be performed by an experienced interventional radiologist [1].

## Case presentation

A 72-year-old female with a history of acute necrotizing pancreatitis due to gallstones underwent transgastric pancreatic necrosectomy, which was complicated by recurrent cholangitis secondary to biliary stricture. The patient eventually required a Roux-En-Y choledochojejunostomy for biliary drainage. Her hospital course was complicated by portal vein thrombosis, and she was started on warfarin for anticoagulation. However, four months after surgery, the patient had three recurrent episodes of gastrointestinal bleeding as evidenced by melena and hematochezia. The cause of bleeding was not identified despite multiple investigations including upper endoscopy, push enteroscopy, colonoscopy, video capsule endoscopy, and tagged red blood cell scan. The patient was instructed to stop taking warfarin due to recurrent gastrointestinal bleeding and despite this, she presented to another institution due to bright red blood per rectum. She was transferred to our tertiary care center for further care.

**Figure 1:**
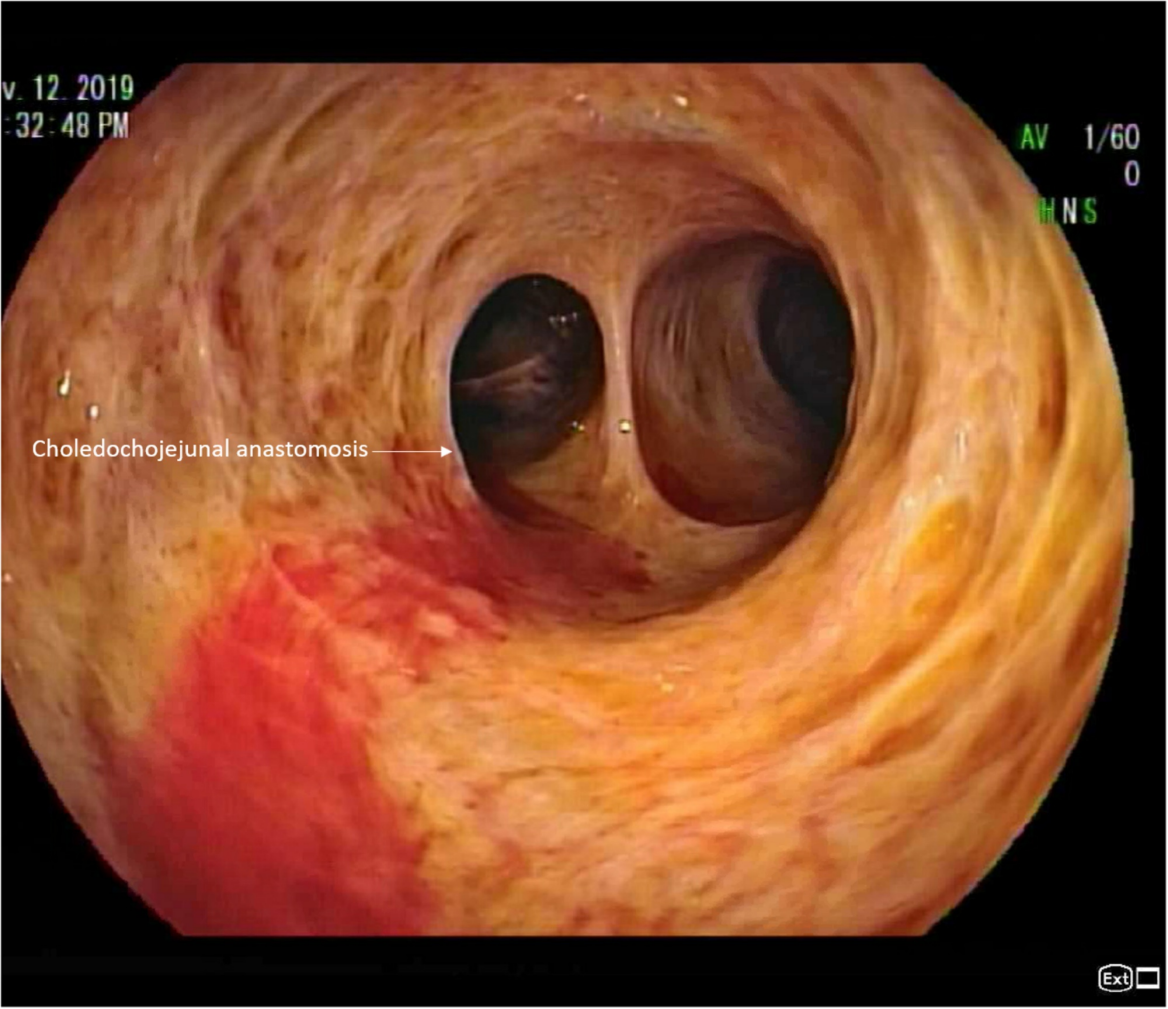
Antegrade double baloon enteroscopy shows hemobilia emerging from the choledochojejunostomy

Upon arrival, she was afebrile with a blood pressure of 96/49 mmHg and a heart rate of 64/min. Her initial blood tests showed a hemoglobin of 6.8 g/dL, a platelet of 83,000/mm^3^, and an international normalized ratio of 1.24. After fluid resuscitation and blood transfusion, the patient underwent a repeat push enteroscopy and colonoscopy without identification of a source of hemorrhage. Due to ongoing bleeding, an antegrade double balloon enteroscopy was performed. The endoscope was traversed through the patient’s roux limb and hemobilia emerging from the choledochojejunostomy was visualized (Figure 1).

**Figure 2:**
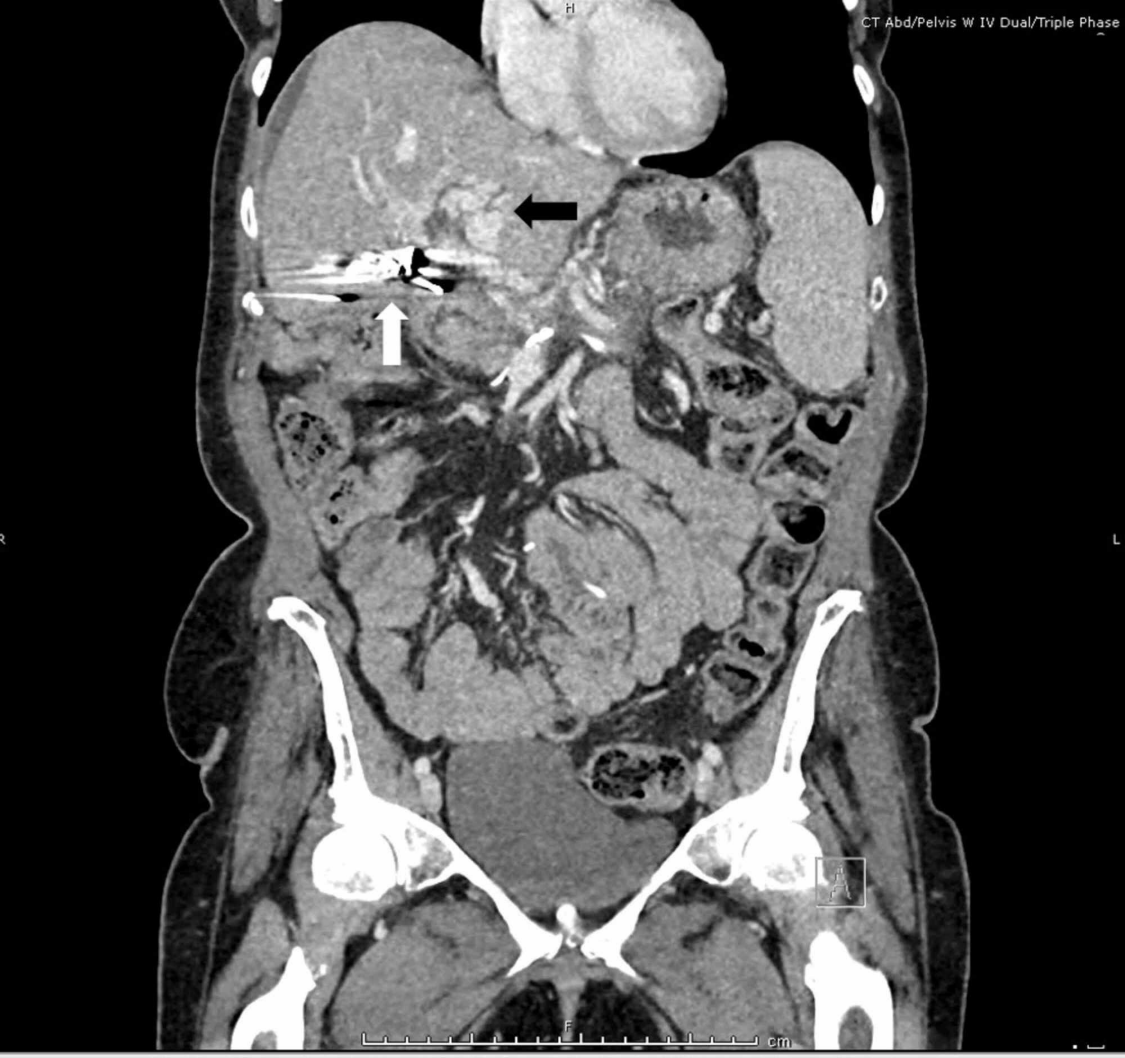
Computed tomography angiography of the abdomen and pelvis shows cavernous transformation (black arrow) and metallic artifact from previous surgery (white arrow) with no active bleeding

Computed tomography angiography of the abdomen showed portal vein and splenic vein thrombosis, with cavernous transformation and no active bleeding (Figure 2). The source of bleeding was thought to be from periportal venous collateral bleeding.

**Figure 3:**
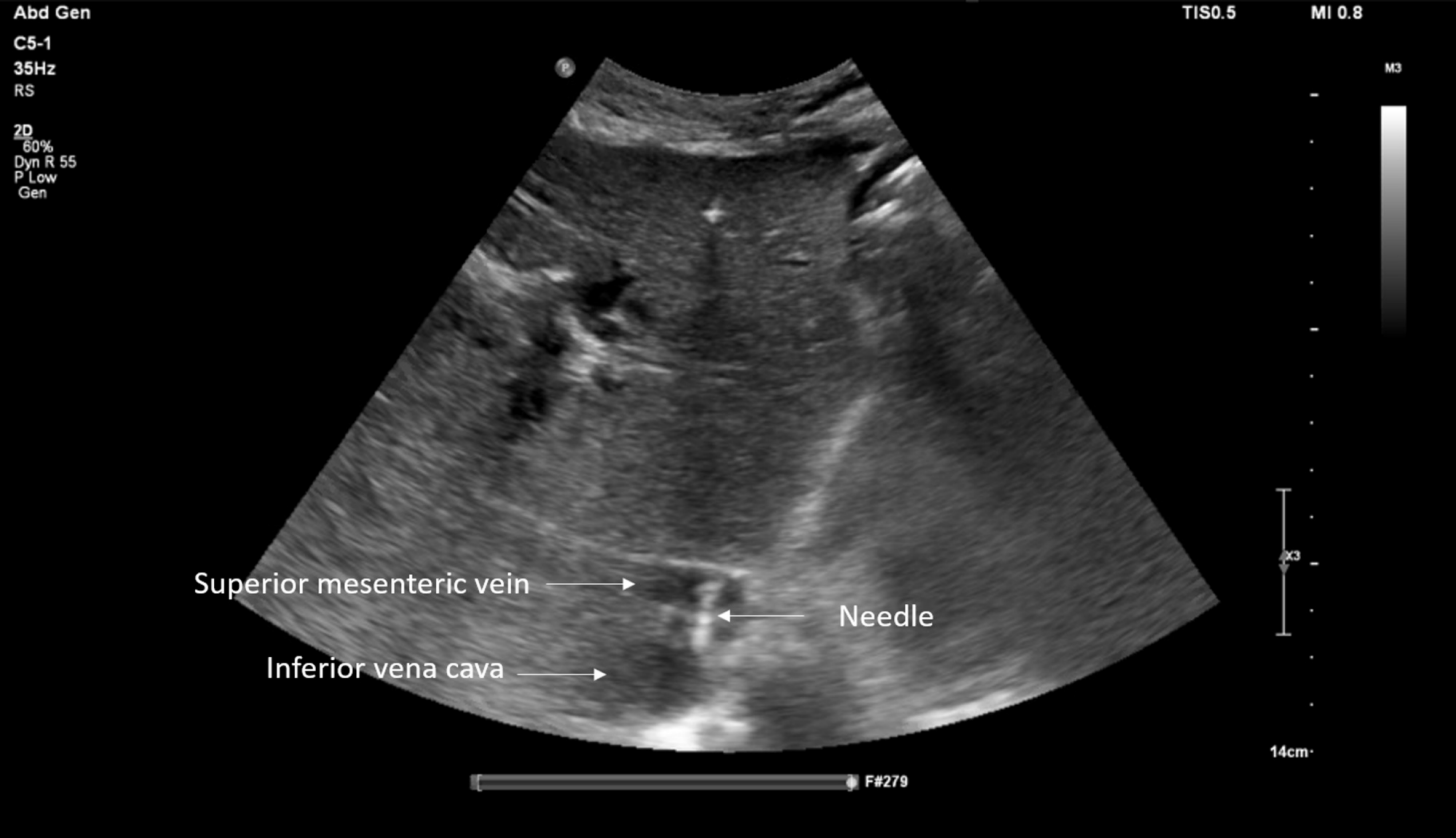
Ultrasonographic finding shows the needle catheter system was passed from the inferior vena cava to the cavernous portal vein branch

**Figure 4:**
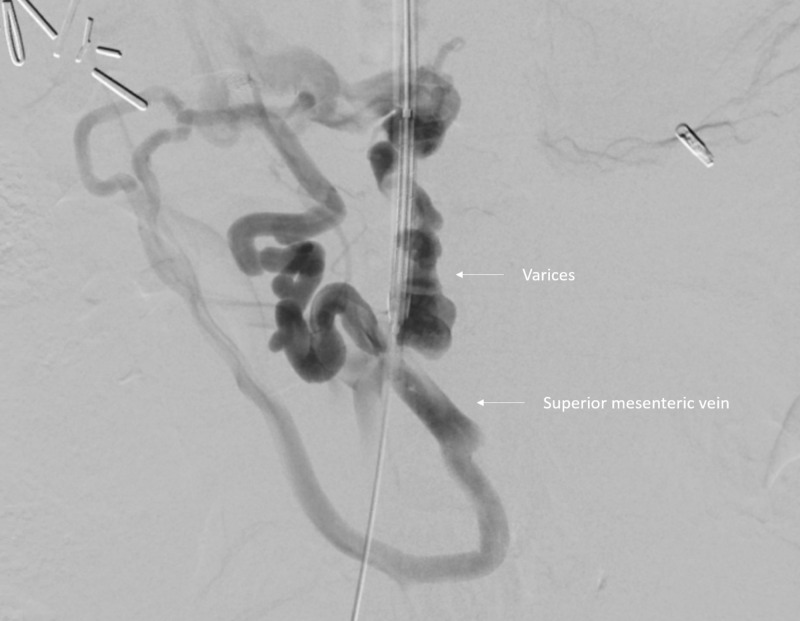
Initial venogram shows cavernous transformation with multiple collateral vessels

**Figure 5:**
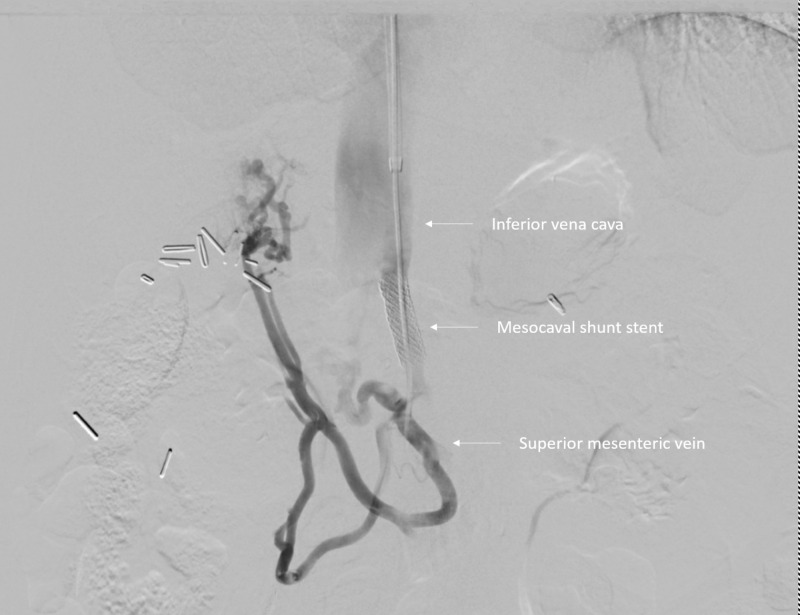
Venogram shows a patent mesocaval shunt with blood flowing from the superior mesenteric vein into the inferior vena cava with decreased collateral flow

Interventional radiology was consulted. Initially, portal vein reconstruction with TIPS was considered but was impossible due to complete occlusion of the portal vein with cavernous transformation and lack of a splenic vein to facilitate reconstruction. Thus, endovascular mesocaval shunt was pursued, connecting the inferior vena cava to a varix that extended from the superior mesenteric vein (Figures 3-5). 

 

 

**Figure 6:**
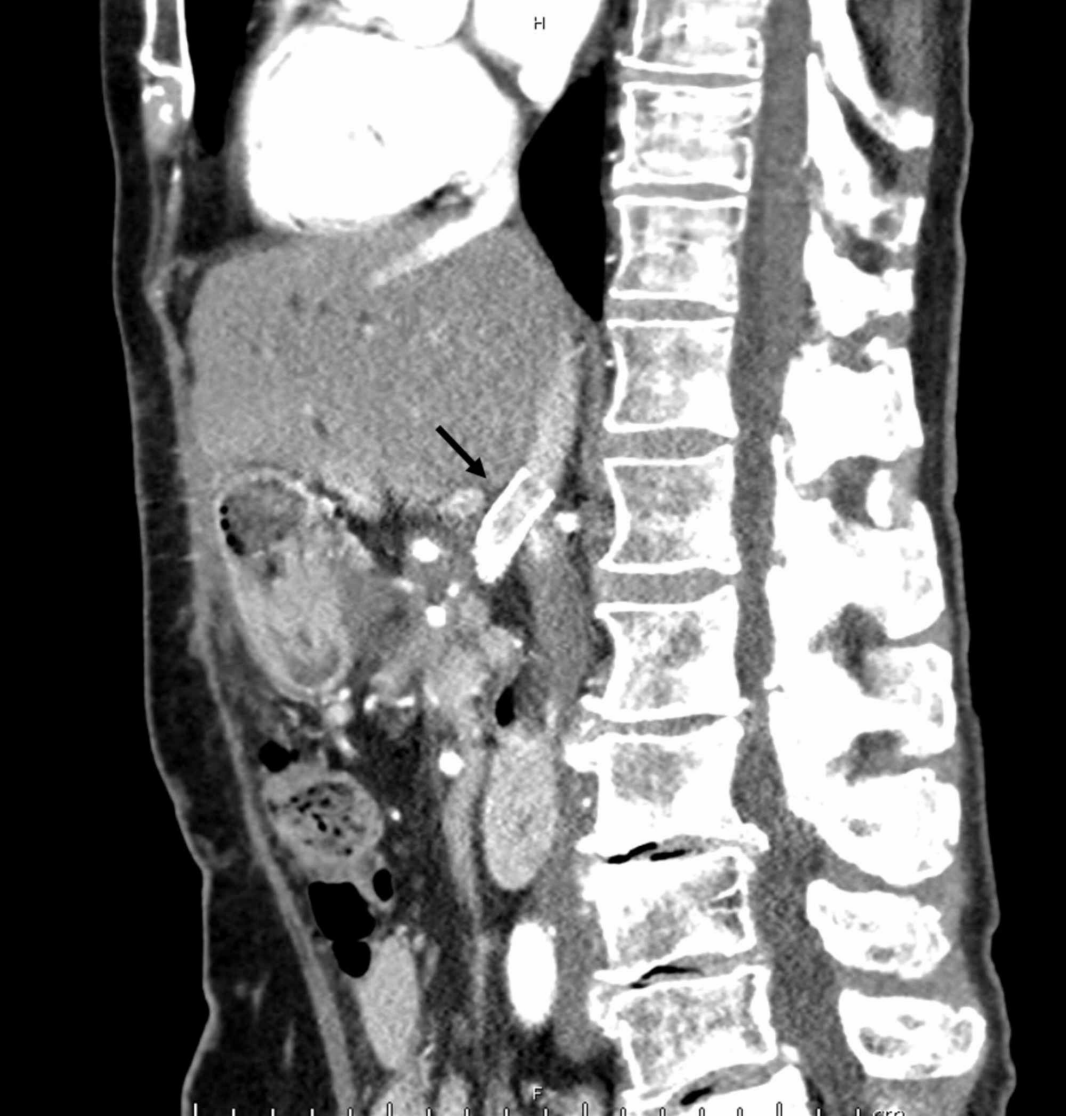
Computed tomography of the abdomen with contrast (sagittal view) shows patent shunt with stent (arrow) between superior mesenteric vein and inferior vena cava

The patient did well after the procedure and has not had any recurrent gastrointestinal bleed at three-month follow up. However, she did develop mild hepatic encephalopathy (HE) that is well controlled with lactulose. The presence of mild HE is consistent with a patent shunt, which was also noted on three-month follow-up imaging (Figure 6). 

## Discussion

Portal hypertension is defined by an increased portal pressure gradient (i.e. the difference in pressure between the portal vein and the inferior vena cava) that is greater than 5 mmHg. However, it becomes clinically significant, with consequences such as ascites and variceal bleeding, when the gradient increases to more than 10 mmHg [2]. Without treatment, the outcome is very poor. The mortality in cirrhotic patients complicated by variceal bleeding is up to 57% at one year [3]. 

In the absence of contraindications, patients with severe complications of portal hypertension, such as variceal hemorrhage and refractory ascites, should be considered for portal system decompression, including percutaneous shunt placement or surgical shunt. TIPS is a common percutaneous shunt placement procedure for portal hypertension, given long track record and clinical experience [1]. However, there are many absolute and relative contraindications, including congestive heart failure, severe pulmonary hypertension, severe coagulopathy, and portal vein thrombosis, such as in our patient [4]. 

Mesocaval shunt creation is an alternative option in patients with chronic portal vein occlusion and can be done surgically or percutaneously. It decreases the portal pressure through an artificial channel connecting the mesenteric vessels to the inferior vena cava, thus bypassing the portal vein [5]. The first percutaneous mesocaval shunt was reported in 1996 by Nyman et al. [6]. In addition to overcoming the anatomical limitation, it also provides the important benefit of preserving a native portal venous anatomy for subsequent liver transplantation [1].

To be eligible for a mesocaval shunt creation, the patient is required to undergo a contrast-enhanced CT to evaluate for anatomical feasibility. Based on previous case reports, six of the nine patients had no recurrent variceal bleeding after the procedure [7]. However, the strength of evidence is limited due to lack of controlled trials. This procedure should be reserved to experienced interventional radiologists, in order to minimize its risks, including hemorrhage due to puncture of proximal vasculature or other organs [5]. Other complications include HE and subsequent shunt occlusion, which require close follow-ups and repeated imaging [5].

## Conclusions

In patients with portal vein thrombosis and portal hypertension, TIPS procedure might not be possible if the portal veins are completely thrombosed. In such cases, mesocaval shunt creation is one of the treatment options to be considered, and can be done surgically or endovascularly by an experienced interventional radiologist.
